# *EGR2* gene mutations: lessons learned from 4 cases

**DOI:** 10.1055/s-0045-1809331

**Published:** 2025-05-26

**Authors:** Wilson Marques

**Affiliations:** 1 Universidade de São Paulo, Faculdade de Medicina de Ribeirão Preto, Ribeirão Preto, SP, Brazil.

Uchoa Cavalcanti et al. (2025) 1 phenotypically and genotypically characterized four cases of a rare form of Charcot–Marie–Tooth disease (CMT) caused by mutations in the *EGR2* gene. Their paper highlights several key features of this gene and its related neuropathies.

First, the heterogeneity of EGR2-associated neuropathies is an important consideration. Although initially described as an autosomal dominant (AD) condition, as seen in two of the patients, it is now recognized that autosomal recessive (AR) cases also occur and that de novo mutations have been reported; these phenomena explain many sporadic presentations. 2

Clinical heterogeneity is also evident. Subject 2 presents with a classic CMT1D phenotype, whereas Subject 1 exhibits Dejerine-Sottas syndrome, and Subject 3 likely has congenital hypomyelinating/amyelinating neuropathy. Subject 4 merits special attention: its motor conduction velocity falls within the intermediate/axonal range, the ulnar compound muscle action potential (CMAP) amplitude is moderately to severely reduced, yet the sensory action potential remains near normal. This combination is unusual for CMT, even when considering late-onset AR axonal neuropathy linked to EGR2 mutations. Could Case 4 represent a predominantly motor intermediate/axonal EGR2 phenotype? On the other side, the distal latencies of median and ulnar CMAP nerves are extremely prolonged, suggesting a preferential involvement of the myelin localized distally, similarly to what is seen in some paraproteinemic neuropathies 3 and sometimes in the hereditary neuropathy with liability to pressure palsy (HNPP). 4 This intriguing question warrants further study.

Respiratory compromise is often neither considered nor investigated in CMT patients, although it can manifest early in the disease course. 5 Comprehensive care of CMT patients should include assessment of respiratory function. Two of the reported patients exhibited significant pulmonary dysfunction, underscoring the need for close monitoring.

These four cases clearly demonstrate the phenotypic variability associated with EGR2 mutations, even among family members carrying the same variant. Such variability is typically attributed to yet-unknown environmental and/or genetic modifiers. Recently, Frezatti et al. (2024) 6 showed that uncommon phenotypes may arise from variants in two different genes, highlighting the additive effects on the final clinical presentation. Although these observations deepen our understanding of phenotypic heterogeneity, definitive answers will only emerge from a more profound elucidation of EGR2's biological function, as emphasized by Uchoa Cavalcanti and colleagues.

Although this paper may appear straightforward at first glance, it underscores the complexity of genotype–phenotype relationships and the importance of a deeper understanding of gene function.

## References

[1] Cavalcanti E BU Santos S CL Couto C M The genetic and clinical spectrum of early growth response 2-related Charcot- Marie-Tooth disease in a Brazilian cohort Arq Neuropsiquiatr 2025 83 04 s00451806820 10.1055/s-0045-1806820 PMC12020502 40262821

[2] NEUROMUSCULAR HOMEPAGE https://neuromuscular.wustl.edu/

[3] Gonçalves T AP Donadel C D Frezatti R SS Monoclonal gammopathy-associated peripheral neuropathies: Uncovering pearls and challenges J Peripher Nerv Syst 2024 29 02 161 172 10.1111/jns.12638 38873841

[4] de Oliveira A P Pereira R C Onofre P T Clinical and neurophysiological features of the hereditary neuropathy with liability to pressure palsy due to the 17p11.2 deletion Arq Neuropsiquiatr 2016 74 02 99 105 10.1590/0004-282 × 20160010 26982985

[5] de Carvalho Alcântara M Nogueira-Barbosa M H Fernandes R M Respiratory dysfunction in Charcot-Marie-Tooth disease type 1A J Neurol 2015 262 05 1164 1171 10.1007/s00415-015-7677-8Erratum in: J Neurol. 2015 May;262(5):1172. doi: 10.1007/s00415-015-7733-4 25761374

[6] Frezatti R SS Tomaselli P J Record C J Overcoming genetic neuromuscular diagnostic pitfalls in a middle-income country Brain Commun 2024 6 06 fcae342 10.1093/braincomms/fcae342 39544699 PMC11562110

